# Synergic Action of Systemic Risedronate and Local Rutherpy in Peri-implantar Repair of Ovariectomized Rats: Biomechanical and Molecular Analysis

**DOI:** 10.3390/ijms242216153

**Published:** 2023-11-10

**Authors:** Bruna Kaori Namba Inoue, Laura Vidoto Paludetto, Naara Gabriela Monteiro, Fábio Roberto de Souza Batista, Igor Lebedenco Kitagawa, Roberto Santana da Silva, Cristina Antoniali, Paulo Noronha Lisboa Filho, Roberta Okamoto

**Affiliations:** 1 Department of Basic Sciences, Araçatuba Dental School, São Paulo State University Júlio de Mesquita Filho—UNESP, Aracatuba 16015-050, SP, Brazil; kaori.namba@unesp.br (B.K.N.I.); laura.paludetto@unesp.br (L.V.P.); naara.monteiro@unesp.br (N.G.M.); fabio.batista@unesp.br (F.R.d.S.B.); cristina.antoniali@unesp.br (C.A.); 2 Federal Institute of Education, Science and Technology of São Paulo (IFSP), Birigui 16201-407, SP, Brazil; igor@ifsp.edu.br; 3 Department of Biomolecular Sciences, Faculty of Pharmaceutical Sciences of Ribeirão Preto, University of São Paulo—USP, Ribeirão Preto 14040-403, SP, Brazil; silva@usp.br; 4 Department of Physics and Meteorology, Bauru Sciences School, São Paulo State University Júlio de Mesquita Filho—UNESP, Bauru 17033-360, SP, Brazil; paulo.lisboa@unesp.br

**Keywords:** osteoporosis, high-fat diet, nitric oxide donor, bisphosphonates

## Abstract

Postmenopausal osteoporosis and poor dietary habits can lead to overweightness and obesity. Bisphosphonates are the first-line treatment for osteoporosis. However, some studies show that they may increase the risk of osteonecrosis of the jaw. Considering the antimicrobial, angiogenic and vasodilatory potential of nitric oxide, this study aims to evaluate the local activity of this substance during the placement of surface-treated implants. Seventy-two Wistar rats were divided into three groups: SHAM (SHAM surgery), OVX + HD (ovariectomy + cafeteria diet), and OVX + HD + RIS (ovariectomy + cafeteria diet + sodium risedronate treatment), which were further subdivided according to the surface treatment of the future implant: CONV (conventional), TE10, or TE100 (TERPY at 10 or 100 μM concentration); *n* = 8 per subgroup. The animals underwent surgery for implant installation in the proximal tibia metaphysis and were euthanized after 28 days. Data obtained from removal torque and RT-PCR (OPG, RANKL, ALP, IBSP and VEGF expression) were subjected to statistical analysis at 5% significance level. For biomechanical analysis, TE10 produced better results in the OVX + HD group (7.4 N/cm, SD = 0.6819). Molecular analysis showed: (1) significant increase in OPG gene expression in OVX groups with TE10; (2) decreased RANKL expression in OVX + HD + RIS compared to OVX + HD; (3) significantly increased expressions of IBSP and VEGF for OVX + HD + RIS TE10. At its lowest concentration, TERPY has the potential to improve peri-implant conditions.

## 1. Introduction

The aging of the world’s population because of improved quality of life and increased life expectancy has raised new concerns about the care of the elderly, who are more prone to the development of various diseases. Osteoporosis is one of the most prevalent chronic diseases in this age group and is characterized by a reduction in bone mineral density and microarchitectural deterioration of this tissue, leading to increased fracture risk due to bone fragility [1,2].

The highest risk group is over 50 years of age, and although there is some incidence in men [3], records from the International Osteoporosis Foundation (2009) indicate a greater predilection for this disease in women, with up to 30% of the female population affected [4]. This is due to the hormonal changes that occur during the postmenopausal period, following a decrease in estrogen levels and an imbalance in bone turnover [5,6], causing resorptive processes to predominate over those of bone formation.

The postmenopausal period may also be associated with a change in the lifestyle of the population, which often becomes sedentary and involves poor dietary habits [7]. During this period, with the loss of the protective role of estrogen and the consequent increase in circulating androgens, the distribution of body fat may also change [8], leading to the development of abdominal obesity and metabolic syndrome [9,10,11].

However, studies on the relationship between obesity and osteoporosis remain controversial [9,12,13,14,15,16]. While some suggest that obesity and overweightness may have a protective effect on bone tissue [17], others suggest that the chronic inflammation induced by the accumulation of lipids due to hypercaloric and/or hyperlypidic diets stimulates the synthesis of pro-inflammatory cytokines (such as TNF-α, IL-1þ, IL-6, CRP, leptin, and adiponectin), leading to bone loss in response to the stimulation of osteoclastic differentiation and consequent bone resorption through the activation of the RANKL/RANK/OPG signaling pathway [18,19,20,21,22,23].

In attempts to improve bone quality, various medications have been administered. Suggested pharmacological therapy for osteoporosis involves the use of drugs classified as antiresorptive or anabolic agents. Indicated as first-line treatment [24,25], bisphosphonates are classified as antiresorptive agents, whose performance is to inhibit the differentiation and activity of osteoclasts [26,27]. Among the bisphosphonates, risedronate is noteworthy for its efficacy in combating bone loss caused by osteoporosis in women during the postmenopausal period [28]. It contributes to increased bone density by reducing the number, viability, and activity of osteoclasts and their precursors [29].

Despite their great antiresorptive potential, the use of bisphosphonates remains controversial because of the increased risk of osteonecrosis of the jaw in long-term treatment [30]. Bone damage has been observed in studies of osteoporotic rats treated with sodium alendronate, where prolonged use of this drug caused damage to the alveolar and peri-implant bone repair processes, with a decrease in bone turnover and a negative impact on the quality of bone produced around implants [31,32,33].

As a result, alternative pharmacological therapies [34], such as raloxifene, calcitonin, and teriparatide [35], among others, have been introduced in the search for results with less damage to peri-implant bone repair. However, even under favorable conditions for bone repair, prolonged systemic treatment with these drugs resulted in more startling adverse effects than those seen with bisphosphonate therapy [36,37,38,39].

While maintaining pharmacological therapy with bisphosphonates, a way to locally improve bone repair and osseointegration processes is sought through implant surface treatment. In the context of the continuous development of substances aimed at optimizing the quality of bone tissue and the referred processes, the use of a nitric oxide donor was proposed. This compound, Rutherpy [Ru(terpy)(bdq)NO+]^3+^ (TERPY), was developed in the Chemical Laboratory of the Faculty of Pharmacy and Biochemistry of Ribeirão Preto/USP. Being a nitrosylated metal compound, TERPY can release nitric oxide enzymatically or without a specific biological target in the presence of vascular tissue and reducing agents or by light irradiation [40,41,42].

Nitric oxide is essential for several body mechanisms, especially due to its antimicrobial, vasodilator, and angiogenic properties and its activity in stimulating collagen production [43]; it also has a modulating role in osteoblastic differentiation [44,45,46], which is essential for bone repair and remodeling in the peri-implant region.

In the search for new methods to minimize the consequences of implant placement in individuals with reduced bone repair capacity, it is necessary to evaluate the effect of surface treatment with TERPY on the maximum repair response of peri-implant bone.

## 2. Results

### 2.1. Clinical Data

**Figure 1:**
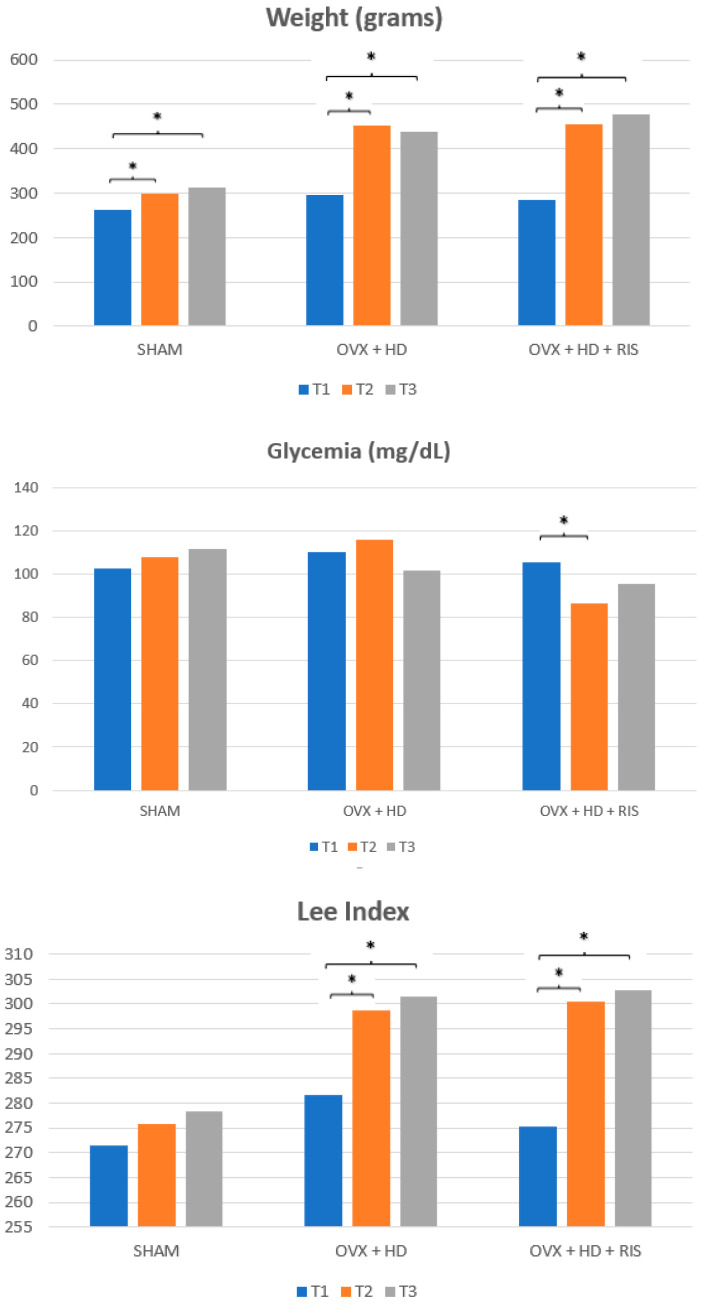
Clinical data graphics of the SHAM, OVX + HD, and OVX + HD + RIS groups during the T1, T2, and T3 periods. *: statistically significant difference.

Regarding the weights of the animals, the three groups showed statistically significant differences (*p* < 0.05) between T1–T2 and T1–T3. For blood glucose, the OVX + HD + RIS group, in the interval between T1 and T2, was the only one with significant differences (*p* < 0.05). For the Lee index, both groups with systemic involvement (OVX + HD and OVX + HD + RIS) showed statistical differences between T1 and T2 and between T1 and T3, as shown in the Figure 1.

### 2.2. Biomechanical Analysis (Counter-Torque)

**Figure 2:**
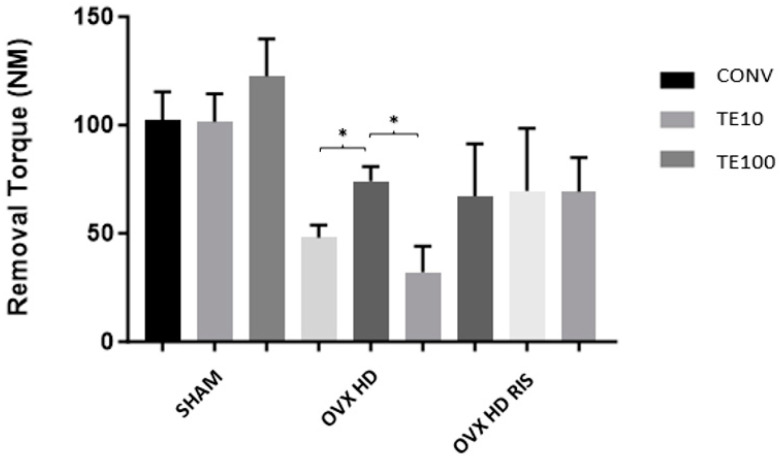
Mean removal torque values between the SHAM, OVX + HD, and OVX + HD + RIS groups. Mean values: SHAM CONV (102.4 N/cm ± 13.0), SHAM TE10 (101.6 N/cm ± 12.7), SHAM TE100 122.4 N/cm ± 17.4), OVX + HD CONV (48 N/cm ± 5.8), OVX + HD TPY10 (74 N/cm ± 6.8), OVX + HD TE100 (32 N/cm ± 12.1), OVX + HD + RIS CONV (67.2 N/cm ± 24.2), OVX + HD + RIS TE10 (69.6 N/cm ± 28.8) e OVX + HD + RIS TE100 (69.2 N/cm ± 15.7). *: statistically significant difference.

In the SHAM and OVX + HD + RIS groups, there was no statistically significant difference between the different implant types. In the OVX + HD group, the TERPY 10 μM (TE10) concentration brought better results, with a statistically significant difference between the CONV-TE10 (*p* < 0.05) and TE10-TE100 (*p* < 0.05) surfaces, as shown in Figure 2.

### 2.3. Molecular Analysis (Real-Time PCR)

**Figure 3:**
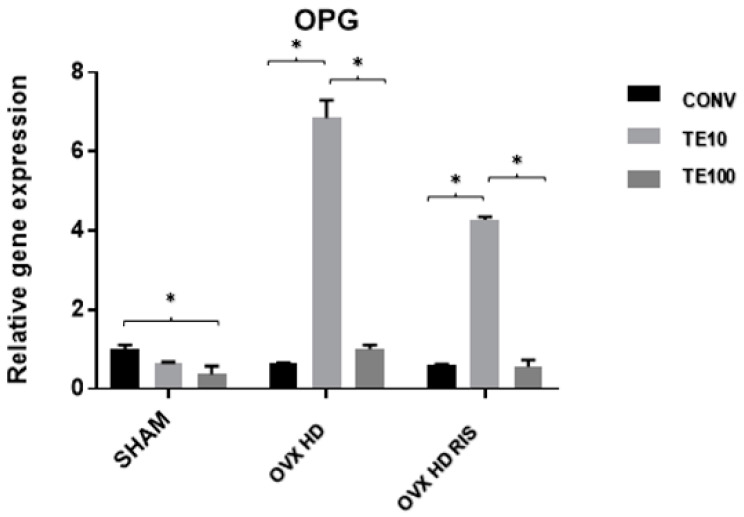
Relative gene expression of the OPG gene for the CONV, TE 10, and TE 100 groups. Mean values of: SHAM CONV (1.000), SHAM TE10 (0.662), SHAM TE100 (0.363), OVX + HD CONV (0.650), OVX + HD TE10 (6.841), OVX + HD TE100 (1), OVX + HD + RIS CONV (0.623), OVX + HD + RIS TE10 (4.271), and OVX + HD + RIS TE100 (0.549). *: statistically significant difference.

In the SHAM group, there was a statistically significant difference in OPG gene expression between the CONV-TE100 surfaces (*p* < 0.05). There were also differences between TE10-CONV and TE10-TE100 in the OVX + HD group, and between TE10-CONV and TE10-TE100 (always with *p* < 0.05) in the OVX + HD + RIS group, as shown in Figure 3.

**Figure 4:**
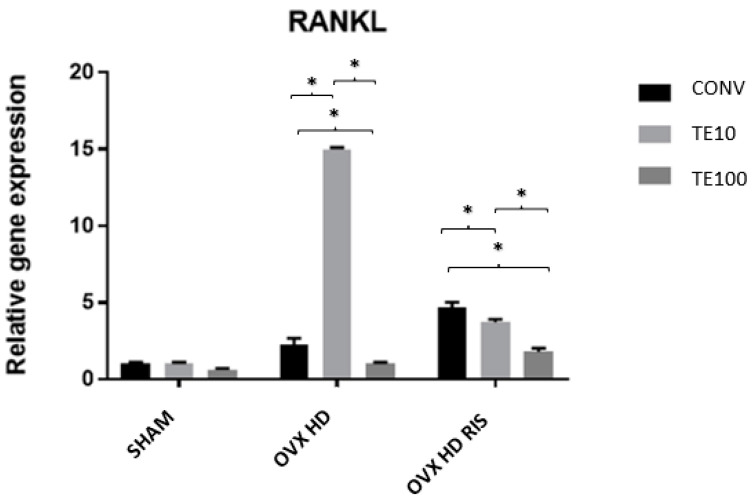
Relative gene expression of the RANKL gene for the CONV, TE 10, and TE 100 groups. Mean values of: SHAM CONV (1.034), SHAM TE10 (1.000), SHAM TE100 (0.637), OVX + HD CONV (2.255), OVX + HD TE10 (15.004), OVX + HD TE100 (1.000), OVX + HD + RIS CONV (4.674), OVX + HD + RIS TE10 (3.722), and OVX + HD + RIS TE100 (1.782). *: statistically significant difference.

For the RANKL gene, the SHAM group showed no statistical difference between the three types of surfaces. However, these differences were noticeable in the OVX + HD and OVX + HD + RIS groups (between CONV-TE10, CONV-TE100, and TE10-TE100, in both situations and always with *p* < 0.05), as shown in Figure 4.

**Figure 5:**
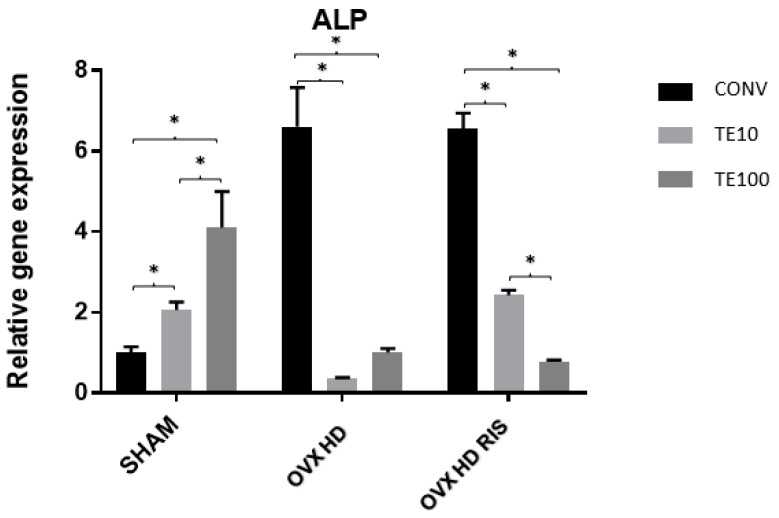
Relative gene expression of the ALP gene for the CONV, TE 10, and TE 100 groups. Mean values of: SHAM CONV (1.010), SHAM TE10 (2.058), SHAM TE100 (4.105), OVX + HD CONV (6.609), OVX + HD TE10 (0.354), OVX + HD TE100 (1.000), OVX + HD + RIS CONV (6.550), OVX + HD + RIS TE10 (2.431), and OVX + HD + RIS TE100 (0.779). *: statistically significant difference.

For the ALP gene, the SHAM and OVX + HD + RIS groups showed statistically significant differences between the CONV-TE10, CONV-TE100, and TE10-TE100 surfaces (*p* < 0.05. As for OVX + HD, the difference applied between CONV-TE10 and CONV-TE100 (*p* < 0.05), as shown in Figure 5.

**Figure 6:**
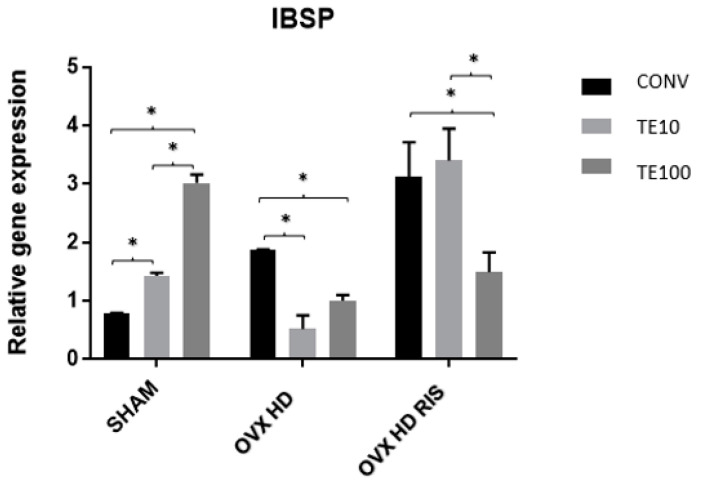
Relative gene expression of the IBSP gene for the CONV, TE 10, and TE 100 groups. Mean values of: SHAM CONV (0.779), SHAM TE10 (1.425), SHAM TE100 (3.017), OVX + HD CONV (1.865), OVX + HD TE10 (0.519), OVX + HD TE100 (1.000), OVX + HD + RIS CONV (3.127), OVX + HD + RIS TE10 (3.400), and OVX + HD + RIS TE100 (1.492). *: statistically significant difference.

For the IBSP gene, the collected data from the SHAM group indicate that there was a statistically significant difference between the CONV-TE10, CONV-TE100, and TE10-TE100 surfaces (*p* < 0.05). In OVX + HD animals, differences can be noted between the CONV-TE10 and CONV-TE100 groups (*p* < 0.05), whereas in OVX + HD + RIS, a statistical difference occurred between CONV-TE100 and TE10-TE100 (*p* < 0.05), as shown in Figure 6.

**Figure 7:**
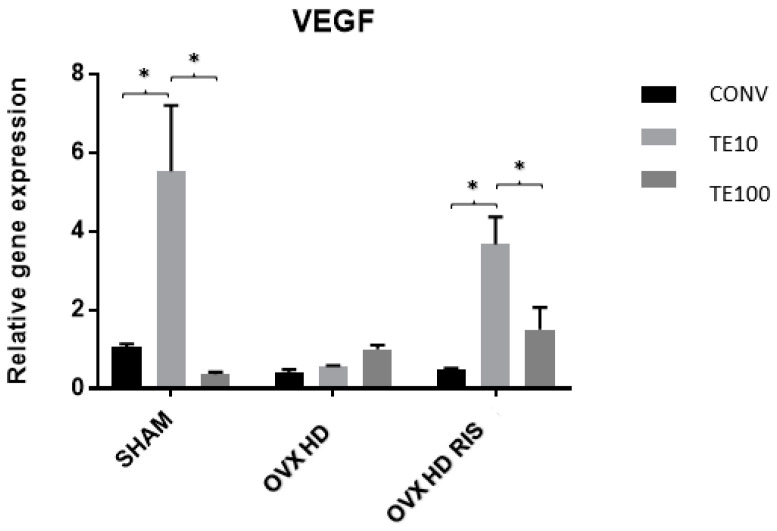
Relative gene expression of the VEGF gene for the CONV, TE 10, and TE 100 groups. Mean values of: SHAM CONV (1.071), SHAM TE10 (5.525), SHAM TE100 (0.360), OVX + HD CONV (0.405), OVX + HD TE10 (0.563), OVX + HD TE100 (1.000), OVX + HD + RIS CONV (0.467), OVX + HD + RIS TE10 (3.666), and OVX + HD + RIS TE100 (1.501). *: statistically significant difference.

In the SHAM and OVX + HD + RIS groups, there were statistically significant differences in relative gene expression for VEGF between the CONV-TE10 and TE-TE100 groups (*p* < 0.05). The OVX + HD group, on the other hand, showed no significant differences, as shown in Figure 7.

## 3. Discussion

In the analysis of the clinical data of the animals, although the three studied groups showed statistically significant differences at the beginning and the end of the experiment (between T1 and T2 and between T2 and T3, with *p* < 0.05), it was observed that the weight gain of the ovariectomized animals was more pronounced: the initial weights of the three groups were in the range of 200 to 250 g, but at the end of the experiment the SHAM group had a weight close to 300 g, while the OVX + HD and OVX + HD + RIS animals exceeded the average of 400 g. There were no major differences between the blood glucose levels of the SHAM and OVX + HD groups during the experiment. Only the OVX + HD + RIS group showed something significant between periods T1 and T2. Finally, the Lee index of the animals brought more remarkable data: only the OVX animals showed statistically significant differences at the end of the experiment (T1–T2 and T1–T3), suggesting an increase in the abdominal circumference of these animals.

The changes in body weight and Lee index of the animals can be explained by the association of the cafeteria diet (hyperlipidemic) with the depletion of estrogen levels resulting from the surgical procedure of ovariectomy, which mimicked the postmenopausal period in humans. During this period, in addition to the loss of the protective role of estrogen on the skeletal system, there is an increase in circulating androgens, leading to changes in body fat distribution that favor an accumulation of abdominal fat [8,47,48,49]. It is worth mentioning that although the animals did not show large variations in their glycemia, insulin resistance is a very common factor observed in overweight or obese people, and if not controlled, can lead to consequences such as type 2 diabetes [18,20,50]. In addition, studies have shown that overweightness in Wistar rats is commonly associated with increased blood pressure, insulin resistance, hyperglycemia, hyperinsulinemia, hyperleptinemia, and dyslipidemia [51]. Most probably, the animals in these studies were suffering from metabolic syndrome, but this cannot be confirmed because lipid profile measurements were not performed; only changes in weight and Lee’s index were observed and documented.

In the biomechanical analysis, whether the surface of the implants was treated made no difference for the SHAM and OVX + HD + RIS groups. Only the OVX + HD group presented a better result for the TERPY 10 μM surface treatment compared to the others. Another pertinent fact emerged when comparing the difference in mean removal torque between the SHAM and ovariectomized groups. The purpose of the removal torque analysis was to evaluate the quality of the osseointegration between bone and implant. In this way, it was observed that this was another point that was affected by the decrease in estrogen production, since the OVX groups had lower mean removal torques. Estrogen deficiency induces changes in the bone by promoting the prevalence of resorptive processes over bone formation, producing more porous bone tissue of lower quality and mineral density [52,53,54,55,56], which results in lower osseointegration, and consequently delaying the bone repair process. The removal torque data of the OVX + HD + RIS group were superior to those of the group without systemic treatment (except for the implant surface treated with TERPY 10 μM), probably due to the mechanism of action of risedronate sodium, which inhibits bone marrow adipogenesis and suppresses RANKL expression, inhibiting osteoclast differentiation and improving bone mineral density [57]. On the other hand, the better results of TE10 surface in the OVX + HD group may be explained by the antimicrobial action of nitric oxide, which is effective against bacterial adhesion to the implant surface and prevents the installation of any local infectious process, allowing better osseointegration [58]. Other properties of nitric oxide are the mediation of inflammatory processes, angiogenesis, and tissue remodeling [44,45,46,59]. In addition, studies have indicated that the antibacterial potential of nitric oxide causes a reduction in postoperative edema and erythema after placement of nitric oxide-releasing pins [60].

For the biomolecular analysis, the relative gene expression of OPG, RANKL, ALP, IBSP, and VEGF was analyzed to characterize the effects of nitric oxide release by TERPY present on the surface of the placed implant. Osteoprotegerin (OPG) and RANKL are genes involved in the dynamics of bone tissue remodeling, indicating cellular activity of osteoblasts and osteoclasts in the tissue through the RANK/RANKL/OPG pathway. In contrast, alkaline phosphatase (ALP) indicates osteoblastic activity and the mineralization process and hydroxyapatite crystal quality, while vascular endothelial growth factor (VEGF) and integrin-binding sialoprotein (IBSP) indicate angiogenic activity (vessel proliferation) and the bone mineralization process, respectively.

Under normal conditions, where there is no estrogen deprivation (SHAM), the relative gene expression of ALP, IBSP, and VEGF was higher in the groups that received implants functionalized with TERPY at both concentrations, suggesting that the compound may favor osteoblastic activity and bone formation processes. Some previous experiments in osteoblast cultures have shown that exogenous nitric oxide can lead to increased osteoblast numbers and osteocalcin production [61,62,63], contributing to bone matrix mineralization.

The TE10 surface promoted increased relative OPG gene expression in OVX + HD animals without necessarily reducing RANKL expression. In OVX + HD + RIS animals, the same increase in OPG was seen, but a reduction in RANKL protein expression was noted. This reaffirms the inhibitory role of bisphosphonates on osteoclastic activity and resorptive processes [28,29], and suggests that TE has an association with OPG expression, promoting osteoblast proliferation and development [61,62]. In the latter group [OVX + HD + RIS TE10], although no increase in ALP was found, significantly increased levels of IBSP and VEGF were found, indicating bone matrix formation and mineralization at these sites.

On the other hand, under the action of TERPY at its lowest concentration, the high gene expression of OPG and RANKL in the OVX + HD group indicated intense cellular activity, both of osteoblasts and osteoclasts, in a constant process of bone remodeling.

The possibility of nitric oxide stimulating the development of osteoblasts was strengthened by the difference between the relative gene expression of OPG and RANKL in the OVX + HD + RIS group, especially under the concentration of 10 μM of TERPY on the implant surface. Together with the action of risedronate sodium, which suppresses bone resorptive processes and osteoclastic activity by inhibiting RANKL synthesis, both variables contributed to the expression of OPG over RANKL, indicating the prevalence of osteoblastic activities over osteoclastic ones.

In groups with at least one of the systemic conditions presented in this study, implants with surfaces treated with TERPY 100 μM did not perform as well as those treated with TERPY at the lowest concentration, suggesting that there may be some level of toxicity when higher concentrations of nitric oxide are present. Studies in cell cultures have shown that the beneficial effects of nitric oxide can be reversed at high concentrations and rapid release rates, potentially leading to apoptosis of these cells [63,64,65,66,67].

## 4. Methods and Materials

### 4.1. Animals

With the approval of the Research Ethics Committee on the Use of Animals of the Faculty of Dentistry of Araçatuba-UNESP (FOA process n° 00563-2018), 72 female rats (Rattus norgicus albinus, Wistar), 3 months old, were used in this study.

Initially, the rats underwent an estrous cycle assessment to ensure that they were cycling normally. For this, the animals were placed in individual cages, and 1 or 2 drops of saline solution were introduced into the vagina daily, which were then aspirated and placed on a histological slide for immediate microscopic reading (Long & Evans technique, 1922) to enable detection of the 4 phases of the estrous cycle. After 2 to 3 regular estrous cycles were observed, the appropriate animals were selected.

The rats were divided into experimental groups according to the type of systemic disorder, diet, and drug treatment (SHAM, OVX + HD and OVX + HD + RIS), and also into subgroups according to the type of implant to be installed (CONV, TERPY 10 μM, and TERPY 100 μM), as explained in Table 1.

### 4.2. High-Fat Diet

The SHAM group received chow (NUVILAB, 1.4% Ca and 0.9% P) and water ad libitum, while the OVX + HD and OVX + HD + RIS groups were fed a high-fat diet (cafeteria diet) throughout the experiment, consisting of daily weighed amounts of stuffed crackers, wafers, and corn chips, totaling 30 g per animal. A bottle of water containing 12% sucrose was also provided, as described in Table 2.

### 4.3. Clinical Data Collection

To follow the development of metabolic changes in the animals, clinical data such as weight, blood glucose, and Lee index of each animal were obtained at 3 different times: before SHAM or bilateral ovariectomy surgery (T1), before the installation of the tibial implants (T2), and before the animals were euthanized (T3).

### 4.4. Fictitious Surgery and Bilateral Ovariectomy Surgery

Rats in the OVX + HD and OVX + HD + RIS groups were anesthetized with 5 mg/kg intramuscular xylazine hydrochloride (Xylazine—Coopers, Paulínia, SP, Brazil, Ltda.) and 50 mg/kg intramuscular ketamine hydrochloride (Injectable Ketamine Hydrochloride, Fort Dodge, Saúde Animal Ltda, Campinas, SP, Brazil.) and then immobilized on a surgical board in lateral decubitus position. A 1 cm incision was made on the flanks, separating the subcutaneous tissue and then the peritoneum to gain access to the abdominal cavity. Once the ovaries and uterine horns were located, they were sutured with Polyglactin 910 4.0 (Vicryltm—Jhonson & Jhonson, New Brunswick, NJ, USA) for subsequent removal of the ovaries. At the end, the suture was made by planes with Polyglactin 910 4.0 thread (Vicryltm—Jhonson & Johnson, New Brunswick, NJ, USA).

SHAM group rats underwent the same procedure, but only to the point of surgical exposure of the uterine horns and ovaries, without their respective laceration and removal.

### 4.5. Systemic and Local Drug Treatments

#### 4.5.1. Systemic Treatment with Risedronate Sodium

Thirty days after SHAM and bilateral ovariectomy, the OVX + HD + RIS group began treatment with risedronate sodium (ApothiMed Ltda., Araçatuba, SP, Brazil) at 35 mg/kg. The drug was diluted in saline solution and administered weekly by gavage using a curved gavage needle (Insight equipment) until the end of the experiment. Animals in the SHAM and OVX + HD groups received the vehicle (saline) only during the same period.

#### 4.5.2. Implant Surface Functionalization with TERPY

The surface functionalization of the TERPY 10 μM and TERPY 100 μM implants was performed using the “layer by layer” technique, under the guidance of Associate Prof. Paulo Noronha Lisboa-Filho. This technique consists in immersing a solid substrate, whose surface has a certain charge imbalance, for a certain time in an aqueous solution containing the material to be deposited. The charge of the material must be opposite to that of the substrate for adsorption by electrostatic attraction to occur. The substrate + monolayer is then washed to remove excess material and dried with compressed air, nitrogen, or a fan, to be immersed in a new solution, now with an opposite charge to the last one. In this way, ultrathin films are formed consisting of cationic and anionic molecular bilayers that are alternately adsorbed. The new substrate + bilayer assembly is then washed and can be dried again.

**Figure 8:**
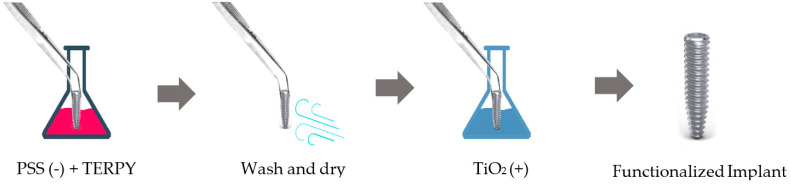
Layer−by−layer technique process.

For the formation of the multilayers, as shown in Figure 8, the sulfonated polystyrene electrolyte (PSS, negatively charged), in which TERPY (concentrations of 10 μM and 100 μM) was dispersed, and a TiO_2_ electrolyte solution (positively charged), previously prepared by the sol-gel method, were used.

The layer-by-layer technique for functionalizing implants is protected by a patent. More information can be found in Section 6.

A total of 48 implants were functionalized, with 24 in 10 μM TERPY and another 24 in 100 μM.

### 4.6. Implant Installation

Thirty days after the start of risedronate sodium administration (60 days after SHAM and bilateral ovariectomy), the animals underwent tibial implantation surgery. Rats were fasted for 8 h prior to surgery and then sedated with a combination of 50 mg/kg intramuscular ketamine (Vetaset—Fort Dodge Saúde Animal Ltda, Campinas, São Paulo, Brazil) and 5 mg/kg xylazine hydrochloride (Dopaser—Laboratório Calier do Brasil Ltda—Osasco, São Paulo, Brazil).

After sedation, the medial portion of the tibia was trichotomized and antisepsis of the region was performed by polyvinylpyrrolidone iodine degerming (PVPI 10%) prior to the incision, which was made with a number 15 bistoury blade measuring 3 cm in length in the proximal tibial metaphysis. The soft tissue was then divulsed in full thickness and pulled apart with the aid of periosteal detachers, exposing the bone to receive the implants.

A total of 72 commercially pure grade IV titanium implants were used, with a diameter of 2 mm and a height of 4 mm, having been surface treated by double acid etching (nitric, hydrofluoric, and sulfuric acids), sterilized by gamma rays, and either subjected or not subjected to surface functionalization with both concentrations of TERPY (10 μM and 100 μM). Milling was performed with a 1.3 mm diameter spiral burr mounted on an electric motor (BLM 600^®^; Driller, São Paulo, SP, Brazil) at a speed of 1000 rpm under irrigation with isotonic 0.9% sodium chloride solution (Fisiológico^®^, Laboratórios Biosintética Ltda^®^, Ribeirão Preto, SP, Brazil) and a 20:1 reduction contra-angle (3624N 1:4 angle piece, 67RIC 1:4 head, KaVo^®^, Kaltenbach & Voigt GmbH & Co., Biberach, Germany) with a depth of 3.0 mm, with initial locking and stability. Installation was performed manually using a digital key.

Each animal received 1 implant in the region of the proximal tibial metaphysis, and later the tissues were sutured in layers using absorbable yarn (Polygalactin 910—Vycrill 4.0) with continuous stitches in the deep plane and monofilament yarn (Nylon 5.0) with interrupted stitches in the outermost plane. In the immediate postoperative period, each animal received a single intramuscular dose of 0.2 mL of penicillin G-benzathine and dipyrone sodium (1 mg/kg/day) for analgesia.

### 4.7. Euthanasia

Animals in the SHAM, OVX + HD, and OVX + HD + RIS groups were euthanized 28 days after the implant installation surgery. With the tibiae in vivo, biomechanical removal torque analysis was performed, or samples were collected from the areas of interest for PCR, which were stored in a −80 °C freezer.

### 4.8. Proposed Analyses

#### 4.8.1. Biomechanical Analysis (Removal Torque)

For removal torque analysis, the implants exposed in the specimens were connected to the digital torque tool. A counterclockwise movement was performed, increasing the removal torque until the implant rotated within the bone tissue and the bone-implant contact was completely broken. The device recorded the maximum peak torque for this fracture in newton-meter (NM), and the values were pooled and subjected to statistical analysis.

#### 4.8.2. Molecular Analysis (Real-Time PCR)

Immediately after removal of the implants from the tibias using reverse torque, the peri-implant bone was collected with mini gouge forceps, maintaining at least 0.5 cm on each side of the peri-implant space and preserving the bone in contact with the implant threads. The bone was carefully washed in phosphate-buffered saline, frozen in liquid nitrogen, and stored at −80 °C to extract the total RNA using a Trizol reagent (Life Technologies: Invitrogen, Carlsbad, CA, USA). After RNA integrity, purity, and concentration analysis, cDNA was created using one µg RNA via a reverse transcriptase reaction (M-MLV reverse transcriptase: Promega Corporation, Madison, WI, USA). Sample cDNA was pipetted onto the array PCR plate with Taqman Fast Advanced Mastermix (Applied Biosystems, Foster City, CA, USA) to detect genes involved in the bone repair process (Taqman Array Fast 96 well plate, Applied Biosystems). Real-time PCR was performed on a Step One Plus real-time PCR detection system (Applied Biosystems) under the following conditions: 50 °C (2 min), 95 °C (10 min), 40 cycles of 95 °C (15 s), and 60 °C (1 min), followed by a standard denaturation curve. The relative gene expression was calculated by referencing the mitochondrial ribosomal protein expression and normalized by the gene expression of tibia fragments undergoing repair during different experimental periods (ΔΔCT method). The assay was performed in quadruplicate, and the genes evaluated were osteoprotegerin (OPG—Tnfrsf11b Rn00563499_m1), receptor activator of nuclear factor kappa B ligand (RANKL—Tnfrsf11 Rn00589289_m1), and alkaline phosphatase (ALP—Rn00564931_m1). Integrin binding sialoprotein (IBSP—Rn00561414_m1) and vascular endothelial growth factor (VEGF—Rn01511602_m1), with Beta-actin (β2-Microglobulin, B2M—Rn00560865_m1) as constitutive, as shown in Table 3.

The values obtained from the proposed analyses were submitted to statistical analysis using GraphPad Prism 7.03 software. The test for homoscedasticity was the Shapiro–Wilk test, and the normal distribution of the data was confirmed for all parameters analyzed. Next, the two-way ANOVA test was performed, considering as factors: systemic condition and time for clinical data; systemic condition and surfaces for counter torque and PCR data. In the sequence, the Tukey post-test was performed with significance level *p* < 0.05, to indicate the differences between the groups.

## 5. Conclusions

At its lowest concentration (10 μM), TERPY seems to have the potential to improve peri-implantar conditions, whether in healthy bone tissue or in bone impaired by systemic changes, such as osteoporosis and changes caused by overweight or obesity. The local action of TERPY seems to have an influence on the increase in OPG levels, suggesting a greater osteoblastic activity, and combined with the inhibitory capacity of bisphosphonates on RANKL, it can be said that the placement of functionalized implants in patients using these drugs seems very promising.

## 6. Patents

BR 10 2021 025891 8 process—“Funcionalização da superfície de implantes com molécula doadora de óxido nítrico através da técnica layer by layer como estratégia para a melhora do processo de reparo periimplantar em ratas”(870210118781 petition).

## Figures and Tables

**Table 1:** Experimental groups and subgroups according to systemic impairment and type of implant to be installed. Each group, as mentioned above, was divided according to systemic impairment, diet, and drug treatment (SHAM, OVX + DH and OVX + DH + RIS), with 24 animals per group. These groups were subdivided into 3 other groups according to the type of implant to be installed (CONV, TERPY 10 μM, and TERPY 100 μM), with 8 rats per subgroup.

Groups	Type of Implant	Description
SHAM	CONV (*n* = 8)	Rats subjected to fictitious ovariectomy surgery
	TERPY 10 μM (*n* = 8)	
	TERPY 100 μM (*n* = 8)	
OVX + HD	CONV (*n* = 8)	Rats subjected to ovariectomy surgery and hypercaloric diet
	TERPY 10 μM (*n* = 8)	
	TERPY 100 μM (*n* = 8)	
OVX + HD + RIS	CONV (*n* = 8)	Rats subjected to ovariectomy surgery and hypercaloric diet and treated with sodium risedronate 0.35 mg/kg
	TERPY 10 μM (*n* = 8)	
	TERPY100 μM (*n* = 8)	

**Table 2:** OVX + HD and OVX + HD + RIS diets during the experiment. Each day during the experiment, each animal received 10 g of Stuffed Cracker, 10 g of Waffer, and 10 g of Corn Chips, and unrestricted access to water with 24 g of Sucrose per 200 mL.

Food	Quantity (g/rat/Day)
Stuffed Cracker	10
Wafer	10
Corn Chips	10
Water + sucrose (12%)	24 g + 200 mL of water

**Table 3:** Taqman probes for Real-Time PCR.

Gene	Gene Name	Identification
OPG	Tnfrsf11b	Rn00563499_m1
RANKL	Tnfrsf11	Rn00589289_m1
ALP	ALPL	Rn00564931_m1
IBSP	IBSP	Rn00561414_m1
VEGF	VEGFA	Rn01511602_m1
B2M	B2M	Rn00560865_m1

## Data Availability

Data is contained within the article.
